# An atomic carbon source for high temperature molecular beam epitaxy of graphene

**DOI:** 10.1038/s41598-017-07021-1

**Published:** 2017-07-26

**Authors:** J. D. Albar, A. Summerfield, T. S. Cheng, A. Davies, E. F. Smith, A. N. Khlobystov, C. J. Mellor, T. Taniguchi, K. Watanabe, C. T. Foxon, L. Eaves, P. H. Beton, S. V. Novikov

**Affiliations:** 1 School of Physics & Astronomy, University of Nottingham, Nottingham, NG7 2RD UK; 2 Nanoscale and microscale research centre (NMRC) and School of Chemistry, University of Nottingham, Nottingham, NG7 2RD UK; 3 The National Institute for Materials Science, Advanced Materials Laboratory, 1-1 Namiki, Tsukuba, Ibaraki 305-0044 Japan

## Abstract

We report the use of a novel atomic carbon source for the molecular beam epitaxy (MBE) of graphene layers on hBN flakes and on sapphire wafers at substrate growth temperatures of ~1400 °C. The source produces a flux of predominantly atomic carbon, which diffuses through the walls of a Joule-heated tantalum tube filled with graphite powder. We demonstrate deposition of carbon on sapphire with carbon deposition rates up to 12 nm/h. Atomic force microscopy measurements reveal the formation of hexagonal moiré patterns when graphene monolayers are grown on hBN flakes. The Raman spectra of the graphene layers grown on hBN and sapphire with the sublimation carbon source and the atomic carbon source are similar, whilst the nature of the carbon aggregates is different - graphitic with the sublimation carbon source and amorphous with the atomic carbon source. At MBE growth temperatures we observe etching of the sapphire wafer surface by the flux from the atomic carbon source, which we have not observed in the MBE growth of graphene with the sublimation carbon source.

## Introduction

The future exploitation of graphene in electronics and optoelectronics is likely to require the production of large area layers with a low density of defects and impurities. Great progress has been made during the last decade in growing graphene by chemical vapour deposition (CVD)^1–4^ and this technique enables the growth of films large enough to use in a number of applications.

The growth of graphene by molecular beam epitaxy (MBE) is also attracting much attention and has been intensively investigated using both gaseous and solid sublimation sources for carbon^5–47^. During the last 7 years graphene layers have been successfully grown by MBE with both types of carbon sources with similar deposition rates and the discussion about the optimum design of MBE source for carbon is still ongoing. Recently, our group demonstrated that epitaxial layers of highly strained graphene can be grown on hexagonal boron-nitride (hBN) using high temperature MBE system^41, 42^. We used a SUKO-63 carbon sublimation source from Dr. Eberl MBE-Komponenten GmbH to grow graphene on hBN flakes and on sapphire at substrate temperatures between 1000 and 1500 °C. Carbon is evaporated by Joule-heating a high-purity graphite filament to sublimation temperatures by passing a large electric current through the filament. Atomic force microscopy (AFM) of graphene grown by MBE on hBN flakes reveals that the graphene layer is highly strained with a hexagonal surface moiré pattern, whose period varies from 13 nm to 30 nm^42^.

It is well-established that high-temperature sublimation of graphite produces a flux containing a mixture of carbon clusters^48–50^. The predominant clusters in the flux are C_1_, C_2_ and C_3_ and clusters larger than C_6_ are negligible. Mass spectrometry studies show that the carbon flux during sublimation of graphite consists mainly of C_3_ (~60%), together with small amounts of C_1_ (~25%) and C_2_ (~15%)^49^. Therefore, in order to achieve epitaxial growth of graphene by MBE from a carbon sublimation sources it is necessary to dissociate C_2_ and C_3_ clusters at the growth surface or incorporate them as dimers or trimers. If C_2_ and C_3_ clusters are not completely dissociated, they may provide a source of carbon deposits on the graphene or substrate surface, and indeed we, and others, have experimentally observed the formation of carbon deposits on the surface of our MBE-grown graphene layers^35, 41, 42^. For these reasons a source of atomic carbon C_1_ may provide significant advantages for the MBE growth of graphene, should such a source become available.

However, results of recent theoretical modelling of the MBE growth process suggested that carbon trimers C_3_ may be beneficial for van der Waals (vdW) epitaxy to produce high quality graphene growth, while atomic carbon deposition is a surface-reaction limited process accompanied by strong chemisorption^51^. Experimental studies are required to test these theoretical results.

More than fifty years ago it was shown that a flux of atomic carbon can be achieved by the evaporation of carbon from a sealed tantalum (Ta) tube^49^. However, it was difficult to avoid co-evaporation of a high flux of Ta atoms and CO molecules. Recently a new design of atomic carbon source has been proposed^52^, in which carbon powder with ^12^C or ^13^C isotopes is contained in a thin-walled (0.05 mm thick) tantalum tube. After sealing, the Ta tube is Joule-heated by a large direct electric current. At about 2000 °C the sublimated carbon reacts with the tantalum cylinder to produce tantalum carbide^49, 52^. The solvated carbon atoms diffuse to the outer surface of the tantalum carbide tube and evaporation of atomic carbon from the surface of the Ta tube takes place^49, 52^. Mass analysis of the carbon species reveals that the flux is predominantly atomic carbon, with very low abundances of C_2_ and C_3_ clusters (<1%)^52^.

This paper reports on the use of this novel atomic carbon source for the MBE growth of graphene layers on hBN flakes and on sapphire wafers.

## Experimental

The growth of graphene was performed using a custom-designed, dual chamber GENxplor MBE system modified by Veeco to achieve growth temperatures of up to 1850 °C in ultra-high vacuum conditions on a rotating substrates of up to 3 inches in diameter. In MBE, the substrate temperature is normally measured with an optical pyrometer; however, in this study we use thermocouple readings because a pyrometer is not compatible with transparent sapphire substrates.

**Figure 1:**
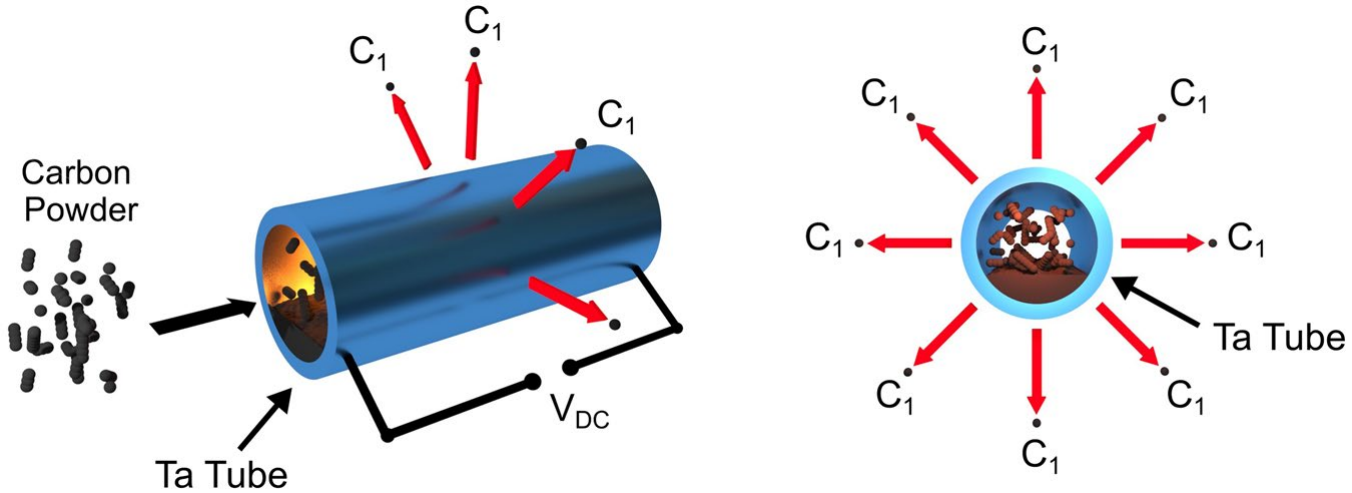
Schematic diagram of the novel atomic carbon source. Carbon powder is sealed into a thin-walled Ta tube, which is subsequently heated by a DC current.

The new atomic carbon source was designed and assembled by Dr. Eberl MBE-Komponenten GmbH. It has been shown to produce predominantly a flux of atomic carbon C_1_ and suppresses C_2_, C_3_ and other high-mass carbon clusters^52^. The source consists of a tantalum foil tube, filled with graphite powder and sealed at both ends. A current of up to 115A heats the tantalum tube to produce a flux of predominantly atomic carbon, as shown schematically in Fig. 1.

We have used this atomic carbon source for MBE growth of graphene layers on hBN flakes exfoliated from high-temperature high-pressure grown bulk hBN crystals^53^ and mounted on sapphire substrates. These are subsequently cleaned using a combination of solvents and annealing with H_2_-Ar gas in a barrel furnace to remove any residual contamination from the exfoliation process. The full procedure for preparing the hBN/sapphire substrates is discussed in our earlier publications^41, 42^. Sapphire wafers did not have any metal coating on the back to avoid any potential contamination during epitaxy at high growth temperatures. We can estimate that the growth surface temperature is about 150–200 °C lower than the MBE heater thermocouple temperatures quoted in our paper. We have also used the existing SUKO-63 as a standard carbon sublimation source for the MBE growth of graphene reference layers.

Images of the grown graphene/hBN heterostructures were acquired with amplitude-modulated tapping (AC) mode AFM (AC-AFM) in repulsive mode under ambient conditions using an Asylum Research Cypher-S AFM and MultiA75AI-G (Budget Sensors, stiffness ~3 N/m) cantilevers. AFM image processing and analysis were performed using Gwyddion and MATLAB software packages. The composition of the grown layers was analysed using X-ray photoelectron spectroscopy (XPS) with a mono-chromated Al Kα X-ray source (1486.6 eV) operated at an emission current of 10 mA and a 12 kV anode potential. Both high sensitivity and wide scan spectra were used to estimate the total atomic concentrations of the detected elements. Raman spectra were obtained with a Horiba–Jobin–Yvon LabRAM Raman microscope using 0.8/100× objective, with a 600 lines/mm grating, a Synapse CCD detector and a laser excitation wavelength of 532 nm, operating at ~4 mW. The Raman shift was calibrated using a Si (001) reference sample.

## Results and Discussions

To test whether the novel source could produce a flux of carbon and to measure the potential carbon deposition rates we first grew carbon layers on unheated sapphire substrates at approximately room temperature. These conditions do not produce graphene, but are expected to result in minimal re-evaporation of carbon from the sapphire during deposition. We observe deposition of carbon on sapphire from the new source and use AFM to measure the thickness of the layer following growth. The carbon layer is weakly attached to the sapphire substrate and can be easily removed by a scraping with gentle pressure using clean plastic-tipped tweezers; this exposes the underlying bare sapphire substrate without damaging it. The thickness is then measured by AFM by scanning the edge of the region where the material has been removed^41^. This method allows us to calibrate the growth rate. For example, a current of 110A through the atomic carbon source produces a carbon deposition rate of ~12 nm/h. This is higher than the carbon deposition rates reported by us for a SUKO-63 sublimation source – for a current of 95A the deposition rate was ~2 nm/h^41^ at a substrate temperature of 1500 °C and it increased to ~4 nm/h for a filament current of 100A. However, we need to remember that we are comparing here with the SUKO-63 deposition rates at the high growth temperature, where we have some re-evaporation of carbon from the growth surface.

**Figure 2:**
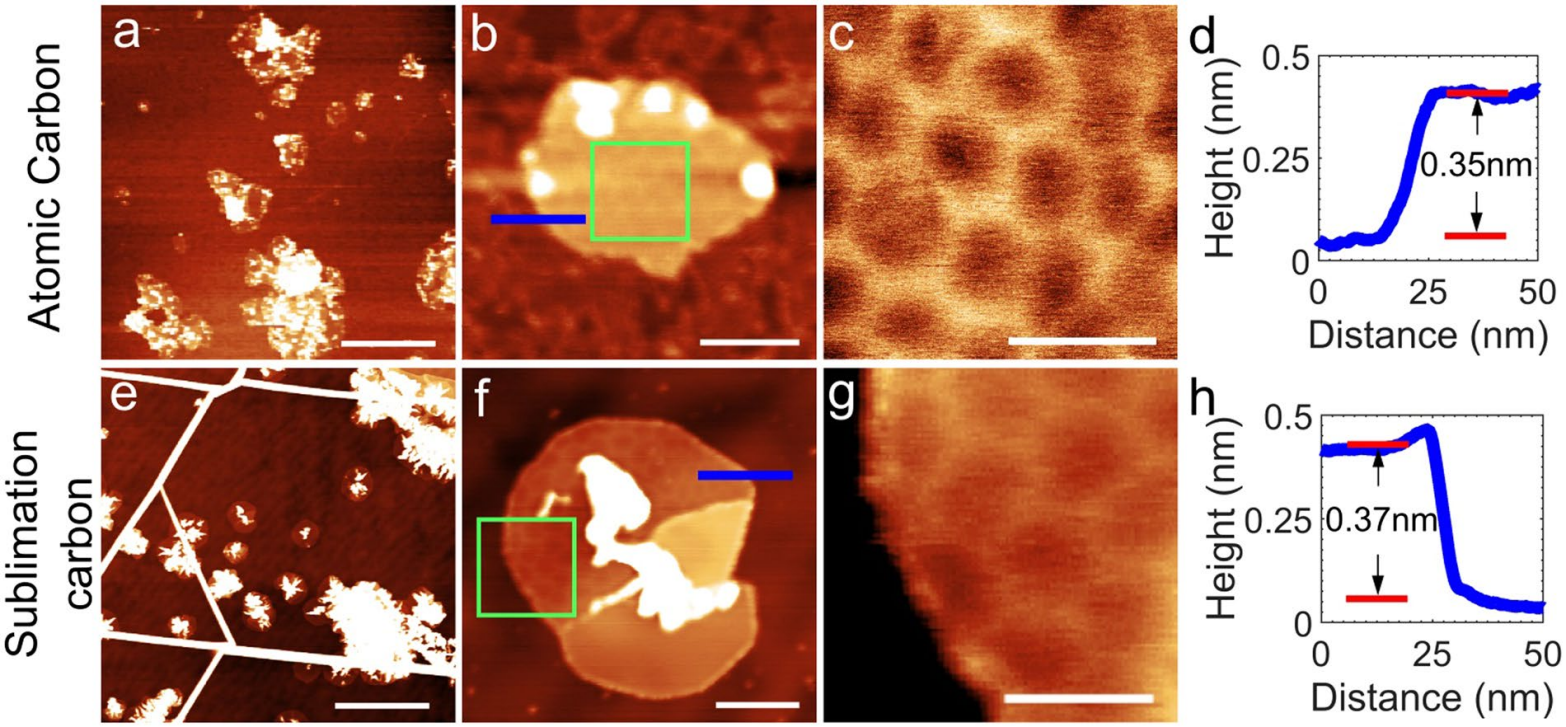
AFM topography images showing the early stages of graphene island nucleation on hBN at a substrate temperature of 1400 °C. Images (**a**–**c**) show graphene islands grown for 5 h using the atomic carbon source and images (**e**–**g**) show graphene grown for 1 h with a carbon sublimation source. Images (**c** and **g**) show the region highlighted by the green boxes in images (**b** and **f**) respectively. The profiles in images (**d** and **h**) show a line profile across the positions indicated by the blue line in figs (**b** and **f**) respectively. The scale bars for the images are as follows; (**a** and **e**) 500 nm, (**b** and **f**) 50 nm, (**c** and **g**) 20 nm.

Following calibration, we grew graphene layers at high temperature of ~1400 °C on the hBN flakes mounted on sapphire substrates. Figure 2 shows AFM images of graphene samples grown on hBN under identical conditions but using the two different carbon sources, the new atomic carbon source (Fig. 2a–d) and the sublimation carbon source SUKO-63 (Fig. 2e–g). Both graphene layers were grown at the same substrate temperatures of approximately 1400 °C, although the growth times differed to ensure a comparable overall coverage of carbon. For MBE with the atomic carbon source we used a filament current of 110A with the total voltage across the cable of ~10 V, which will result in a power of ~1.1 kW. In the case of MBE with the SUKO-63 carbon source a current of 100 A will result in a voltage of ~14 V and a power of ~1.4 kW.

**Figure 3:**
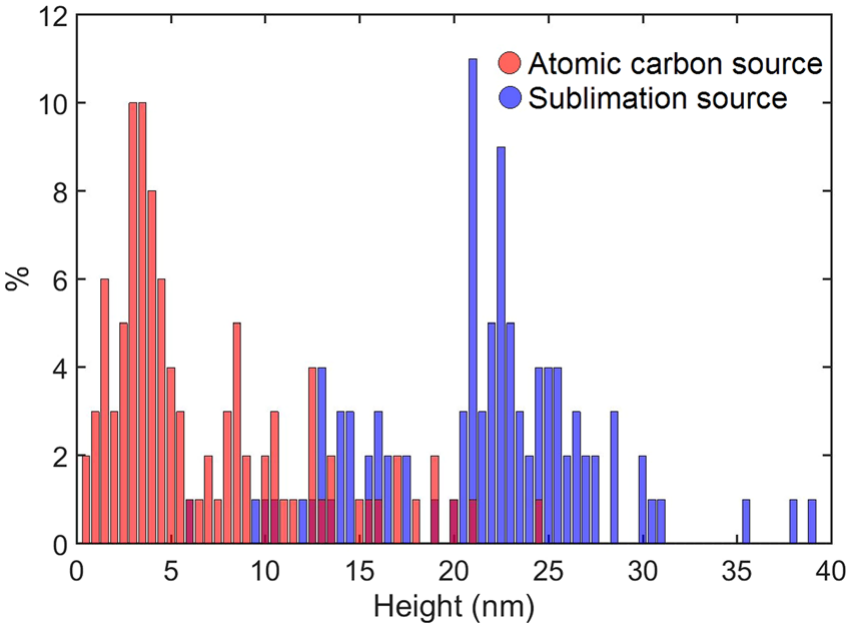
Height distribution of bulk carbon deposits on the hBN surface for the samples shown in Fig. 2 grown using the atomic carbon source (red) and the sublimation carbon source (blue).

The morphology of the two films grown with different carbons sources show some similarities, but also some significant differences. For the large area images we observe, in both cases, topographically high carbon clusters co-existing with small graphene islands which are shown at higher magnification in images Fig. 2b and f. The lines running across the image in Fig. 2e are due to thermal cycling of hBN flake and are not associated with deposition of carbon^42^. Although the lateral dimensions of the clusters in the large area images are rather similar, their topographic heights are quite different – see Fig. 3, in which a histogram of cluster heights is presented. This shows a wide range of islands, with heights of 5–25 nm, for the sublimation carbon source, but much lower height clusters for the atomic carbon source – typically ~3 nm. The data used for this histogram was extracted by manually measuring the maximum height, with respect to the hBN substrate, of a hundred carbon deposits on each sample (deposits on steps or wrinkles on hBN were not included in the histogram). Measuring the height across the edge of the graphene islands in Fig. 2b and f both provide evidence for monolayer height graphene (as determined from the profiles in Fig. 2d and h). Furthermore, a moiré pattern is observed on both islands (shown more clearly in the zoomed images in Fig. 2c and g) with a period ranging from 13–15 nm, close to the value expected for unstrained graphene which is orientationally aligned with respect to the underlying hBN^54–56^.

One significant difference between the graphene islands grown with the two sources is the morphology of the centres of the islands. For the atomic carbon source, islands of graphene with dimensions ~100 nm width are observed and carbon deposits with lateral dimensions ~20 nm across and ~3 nm high, which tend to be located at the edge of the islands. Typical AFM images of graphene layers grown on hBN are presented in the Supplementary Information (SI). In contrast, the graphene layers grown with the sublimation carbon source typically have a carbon deposit at their centre, suggesting that these deposits may act as a nucleation site for lateral graphene growth. Moreover, these deposits have much larger dimensions, ranging between 5 and 25 nm in height and widths from 50 nm up to as much as several 100 nm in some cases. Thus the AFM results indicate that the new atomic carbon source allows MBE growth of orientationally aligned graphene layers on hBN with significantly smaller carbon deposits than those for the sublimation source we have previously used for MBE graphene growth.

**Figure 4:**
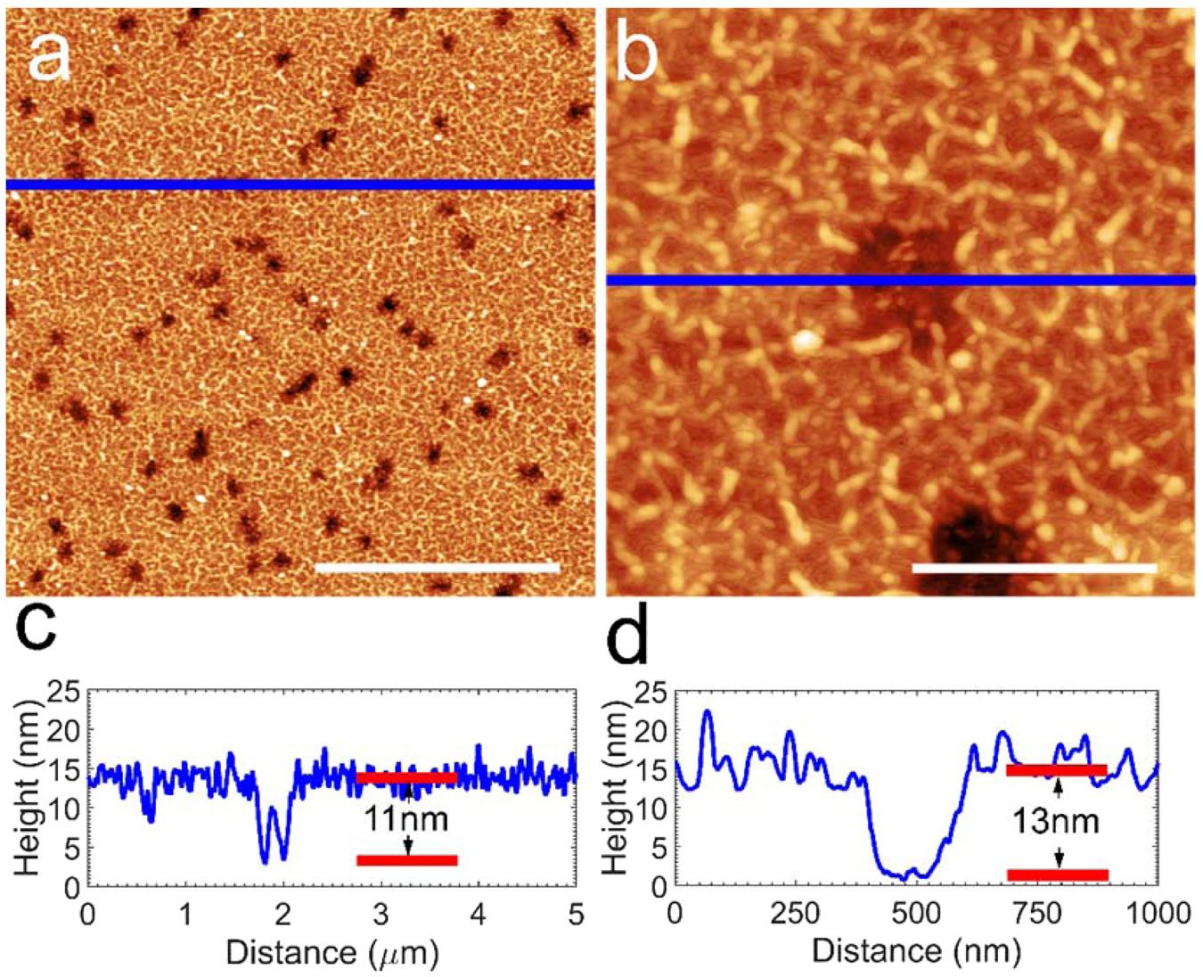
(**a**) AFM image of carbon deposited on sapphire after growth at 1400 °C for 5 h using the atomic carbon source. Scale bar: 2 µm. The surface exhibits a series of pits caused by atomic carbon etching. (**b**) Zoom-in image of the area shown in (**a**). Scale bar: 400 nm. (**c**) Line profile taken across the region indicated by the blue line in (**a**). (**d**) Line profile taken across the region indicated by the blue line in (**b**).

AFM studies of carbon layers grown at ~1400 °C on the uncovered surfaces of the sapphire show rough surfaces for both types of carbon source. However, there is a significant difference between the growth on sapphire with the sublimation and atomic carbon sources. With the atomic carbon source we observes deposition of carbon and the simultaneous development of etch pits on the sapphire surface. Figure 4 shows AFM topography images of the surface between the hBN flakes where carbon impinges directly on sapphire. In these regions there are several ~100 nm wide and ~10 nm deep pits (see profiles in Fig. 4c and d). Previously, with the sublimation carbon source, we observed no significant etching of sapphire by the carbon flux, but instead found a higher deposition rate of carbon on sapphire than on the surface of hBN flakes^41, 42^. Etching of sapphire by a carbon flux has been reported previously for graphene MBE with a sublimation carbon source mounted very close to the sapphire surface^34^. Our data suggest that the flux of atomic carbon from the atomic carbon source is significantly more chemically reactive at MBE growth temperatures of ~1400 °C. This is consistent with previous observations that atomic carbon reacts chemically with the oxygen in sapphire and probably forms CO in a process known as the carbothermic reduction of sapphire at reduced pressures and at high temperatures above ~1000 °C^34, 57^. This is also consistent with the fact that we experimentally observed no etching of sapphire with the atomic carbon source at lower or room temperature MBE growth. AFM images of the carbon layer grown on sapphire with the atomic carbon source at a room substrate temperature and the sapphire surface where the carbon has been removed after the growth are given in the SI.

**Figure 5:**
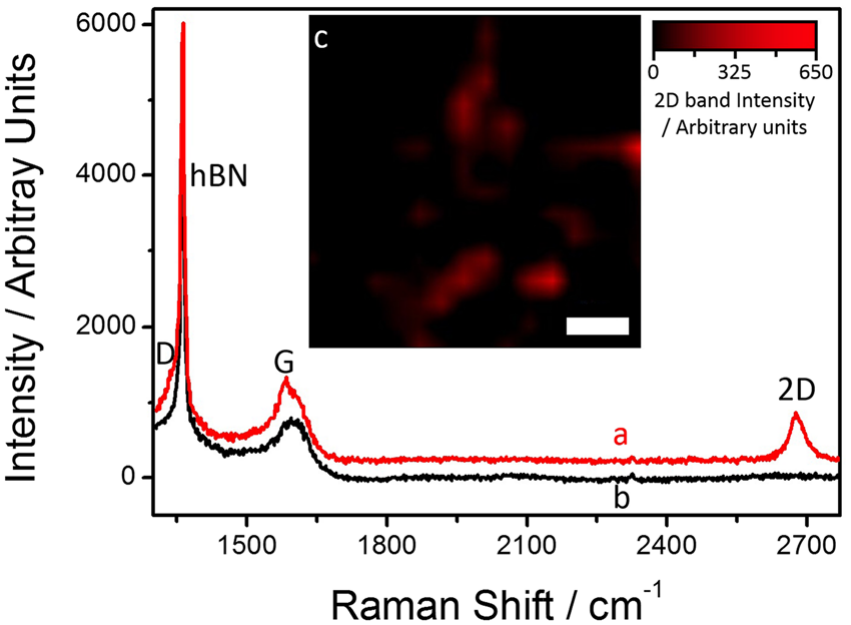
Raman spectra of the graphene layers grown at ~1400 °C on hBN flakes with a sample growth time of 3 h (**a**,**b**). The inset (**c**) shows a Raman map of the intensity of the 2D band for this growth time, with red regions show a Raman spectrum similar to (**a**), whilst darker regions show spectra similar to (**b**). The scale bar in the inset image is 3 µm.

Figure 5(a,b) shows Raman spectra of the carbon layers grown at ~1400 °C on hBN flakes using the atomic carbon source with a 3 hour growth time. Broad G and D bands, at 1591 and 1336 cm^−1^ respectively, were observed across the whole region imaged, as shown in Fig. 5(b). The sharp peak at 1365 cm^−1^ is due to the E_2g_ phonon from the hBN flake^58^. In some areas on the hBN surface, a second carbon related component was observed comprising a 2D band at 2676 cm^−1^, along with a sharp shoulder on the broader amorphous band at 1581 cm^−1^, see Fig. 5(a) and (c). This is similar to our previous reports on graphene grown on hBN using the sublimation carbon source^41, 42^, the component with a 2D band can be assigned to either graphitic carbon aggregates or unstrained islands of graphene. The other component, observed in every Raman spectrum recorded on the material grown on the hBN flakes, consists of a G peak that is significantly broader using the atomic carbon source than the sublimation carbon source (FWHM = 76 and 35 cm^−1^, respectively, a comparison of the G peaks for the two carbon sources is given in the SI), suggesting that the nature of the aggregate formed by the two sources is different - amorphous and graphitic^42^, respectively.

Raman studies of the carbon layers grown on sapphire with the atomic carbon source have demonstrated spectra consistent with the growth of turbostratic graphene (Raman spectra recorded on the material grown on sapphire with both carbon sources are included in the SI). The relative intensities and shape of the Raman spectra were very similar to Raman data from the carbon layers grown on sapphire with the carbon sublimation source^41, 42^.

**Figure 6:**
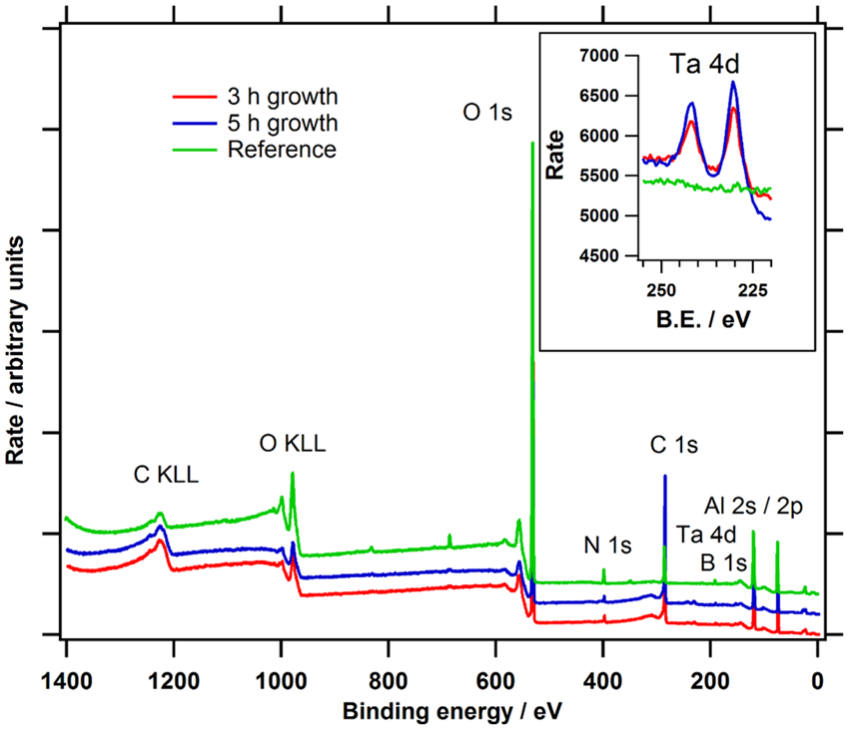
XPS wide scan spectra over a full wide energy range for two graphene samples grown for 3 h and 5 h and one reference sample. The inset is the Ta 4d high sensitivity spectra for the same three sample areas, showing the presence of Ta on all except the reference sample.

Figure 6 shows wide scan XPS spectra over the full energy range for two graphene samples grown with the atomic carbon source and one reference sample. A reference hBN/sapphire wafer was heated in the MBE chamber up to growth temperatures in vacuum, but with no atomic carbon flux. XPS detects the elements in the uppermost 10 nm of a surface and Al, O, C, B, and N are detected on all samples. The XPS tantalum (Ta) signal was observed on all studied graphene layers grown with the atomic carbon source. The inset is a high sensitivity spectrum for each sample over the Ta 4d spectral region, clearly showing the Ta doublet at 231.0 and 242.5 eV on the surface of all graphene samples except for the reference wafer. The Ta concentration was in the range 0.15 to 0.27 atomic %. We are now working to establish the correlation between the Ta concentration and the MBE growth conditions. The effect of Ta-doping on the electrical properties of graphene is also a topic of interest. We have also analysed graphene layers grown with the carbon sublimation source, where we observed no Ta incorporation on the level of sensitivity of our XPS system.

XP spectra for the C 1 s, B 1 s and N 1 s energy regions for the 3 h, 5 h and reference samples are presented in the SI. Carbon is detected on the reference sample, but the intensity is lower and peak shape is significantly different to the 3 h and 5 h deposited samples. The C 1 s peaks appear at ~284.5 eV and are asymmetric to the high binding energy side, with a long ‘tail’ to high binding energy, which is consistent with graphitic/graphene-like bonding. (sp2 bonding), whilst the detected carbon peak on the reference sample is at ~284.7 eV and more symmetrical. The broader peak suggests sp3 bonding dominates in this case. (For this comparison the spectra were further charge corrected to the N 1 s substrate peak at ~397.8 eV.) We propose that it is possible that some adventitious carbon exists on the surface of the reference sample prior to heating and this has reacted or stayed adsorbed onto the sapphire and/or the hBN surfaces. Since all the samples were transferred to the XPS instrument in air after heating and cooling, they may also have additional adventitious carbon, which has deposited during transfer and storage over a few days. Considering the XP spectra for the B 1 s and N 1 s (in SI) we observe that the peak intensity of both B 1 s and N 1 s is higher for the reference sample, which would be expected. No clear changes occur in the peak shape after deposition, so it is not possible to deduce from these spectra if the graphene deposited actually reacts with the hBN substrate.

Our work shows that it is possible to form graphene on hBN flakes by MBE using the new atomic carbon source. We are now performing more studies on the epitaxy of graphene with both atomic and standard sublimation carbon sources to establish the influence of the source design on the properties of graphene layers and results will be published in due course.

## Summary

The growth of graphene by molecular beam epitaxy using different carbon sources is attracting attention as a means of producing high-quality graphene layers. It is well-established that high-temperature sublimation of graphite produces a flux containing a mixture of carbon clusters including C_1_, C_2_ and C_3_. A novel atomic carbon source was used to grow graphene layers by MBE on hBN and sapphire at substrate temperatures of ~1400 °C. Our AFM measurements reveal the formation of hexagonal moiré patterns on the surface of graphene monolayers on hBN flakes. The amount of non-graphene carbon on the surface is reduced for the layers grown with the atomic carbon source when compared with a carbon sublimation source. The Raman signal of the graphene layers grown with the sublimation carbon and the atomic carbon sources on hBN and sapphire are similar, whilst the nature of the carbon aggregates is different - graphitic with the sublimation carbon source and amorphous with the atomic carbon source. Unintentional Ta incorporation to the layers grown with the atomic carbon source need to be further investigated. At MBE growth temperatures we observe etching of the sapphire wafer surface by the flux from the atomic carbon source, which we have not observed previously in the MBE of graphene using a sublimation carbon source. Overall our work shows that it is possible to form graphene/BN heterostructures by MBE using this new atomic carbon source. However, further experimental studies involving both atomic and standard sublimation carbon sources are required to establish which carbon clusters C_1_ or C_3_ are more beneficial for the growth of high quality graphene layers by MBE.

### Data availability statement

The datasets generated during and/or analysed during the current study are available from the corresponding author on reasonable request.

## Electronic supplementary material

Supplementary Information
