# Supplementary material for: An atomic carbon source for high temperature molecular beam epitaxy of graphene
Source: Sci Rep. 2017 Jul 26;7:6598. doi: 10.1038/s41598-017-07021-1 (PMC5529545; doi:10.1038/s41598-017-07021-1)
Supplement: Supplementary file 1 — Supplementary Information [file 41598_2017_7021_MOESM1_ESM.pdf]

An atomic carbon source for high temperature molecular beam epitaxy of graphene.

Supplementary Information

J. D. Albar[1], A. Summerfield[1], T.S. Cheng[1], A. Davies[1,2], E.F. Smith[2], A.N. Khlobystov[2], C.J. Mellor[1], T. Taniguchi[3], K. Watanabe[3], C.T. Foxon[1], L. Eaves[1], P.H. Beton[1], S.V. Novikov[1,*]

[1]School of Physics & Astronomy, University of Nottingham, Nottingham NG7 2RD, UK

[2]Nanoscale and microscale research centre (NMRC) and School of Chemistry, University of Nottingham, Nottingham NG7 2RD, UK.

[3]The National Institute for Materials Science, Advanced Materials Laboratory, 1-1 Namiki, Tsukuba, Ibaraki 305-0044, Japan

* Corresponding author. Sergei.Novikov@Nottingham.ac.uk (Sergei Novikov); Phone - +44-115-9515138

Atomic force microscopy

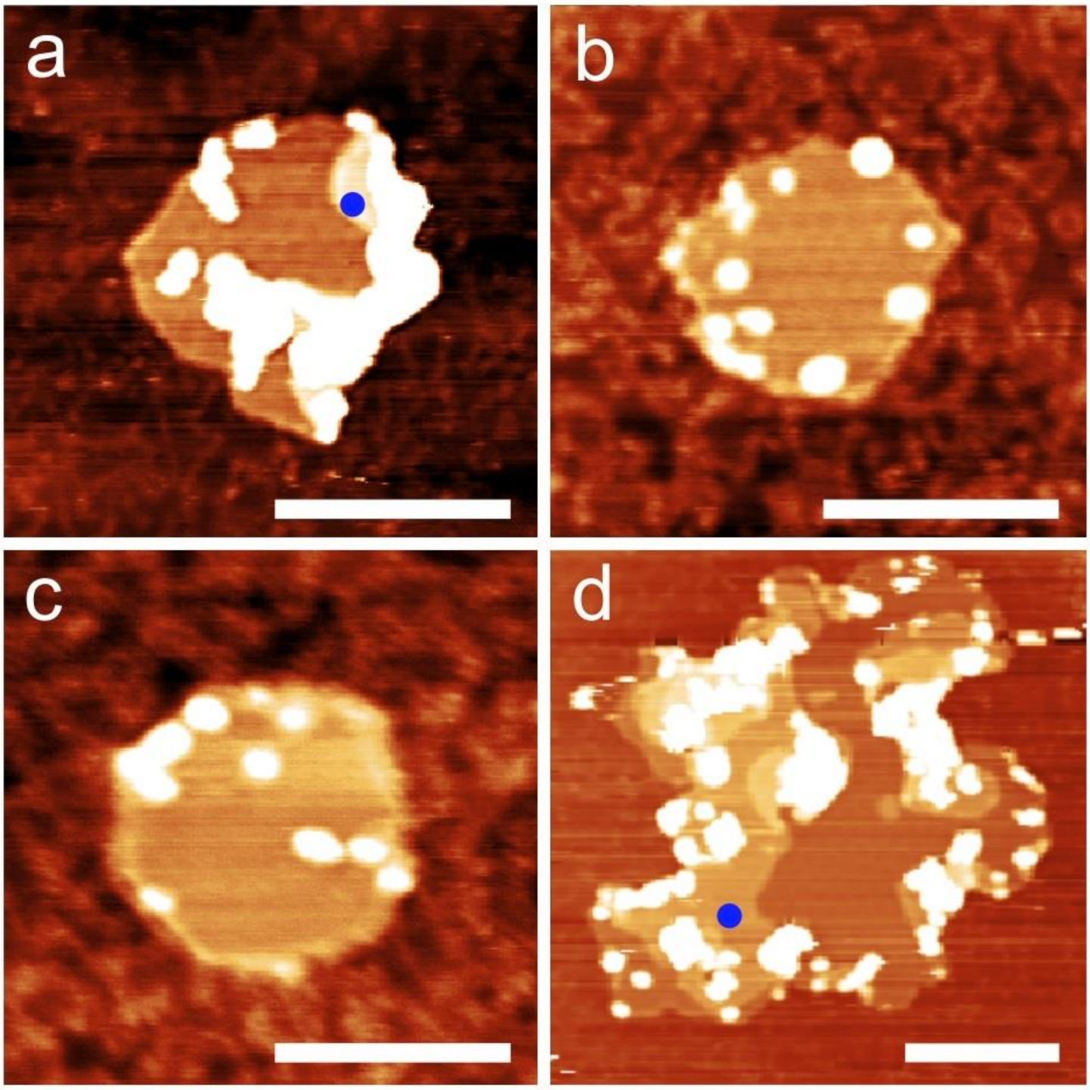

Figure SI-1. AFM topography images of graphene layers grown on hBN with the atomic carbon source at a substrate temperature of ~1400°C showing moiré patterns with ~14 nm period. The carbon deposits tend to be more scattered across the graphene layers compared to those grown with the sublimation source, where they are more clustered around the centre of the layer. Some islands present graphene double layers (see areas indicated by a blue dots on fig. SI-1.a and fig. SI-1.d). All scale bars are 100 nm.

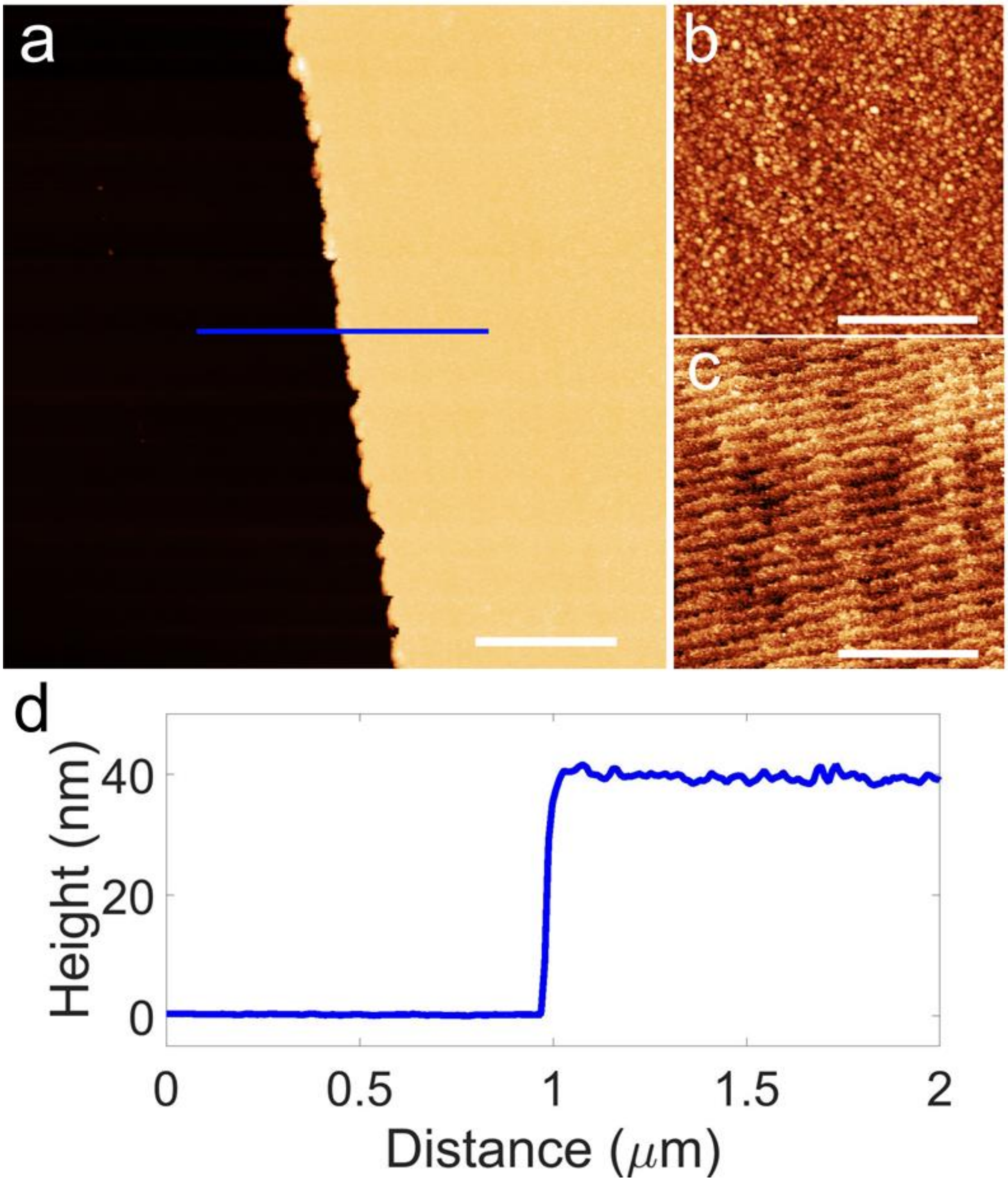

Figure SI-2. AFM topography images of the carbon layer grown on sapphire with the atomic carbon source at a room substrate temperature. a) Edge of a scratch on sapphire done with a pair of tweezers. Scale bar is 1 µm. b) Zoom into the area covered in carbon (right side of the scratch). Scale bar is 400 nm c) Zoom into the uncovered area with the sapphire substrate exposed (left side of the scratch). Scale bar is 400 nm d) Profile along the path indicated by the blue line in (a) showing a carbon thickness of ~40 nm.

Raman data

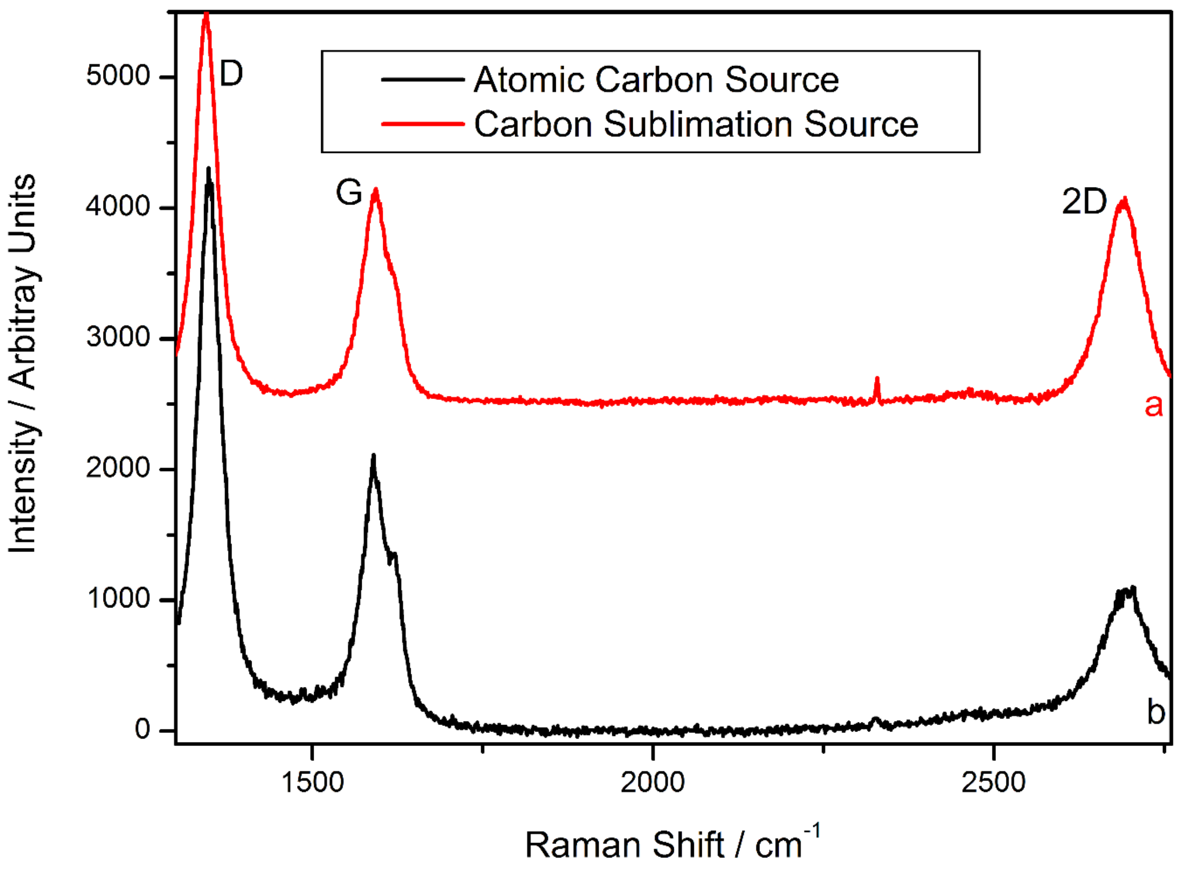

Figure SI-3. Raman spectra of the carbon material grown on sapphire using the atomic carbon source (a) and for comparison the sublimation SUKO-63 carbon source (b).

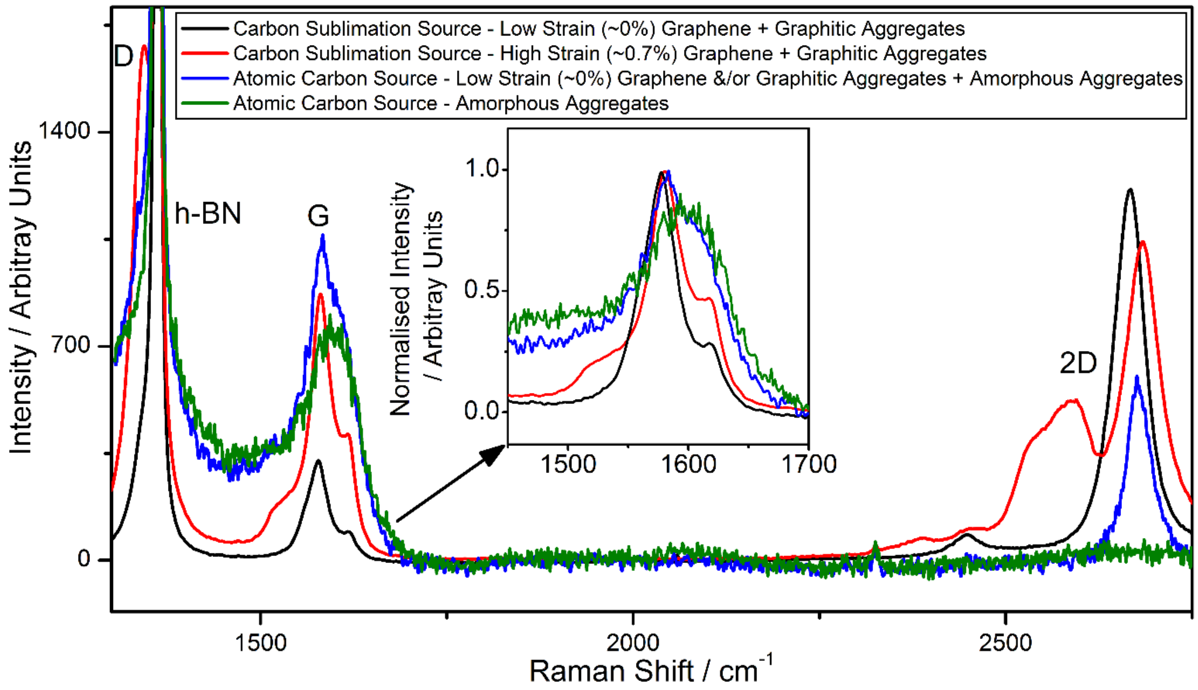

Figure SI-4. Comparison of Raman spectra on the carbon material deposited on hBN. The sublimation carbon source (red & black) is replicated from data published in [43] for comparison to the atomic carbon source (blue & green). Inset: G region showing normalised spectra.

X-ray photoelectron spectroscopy

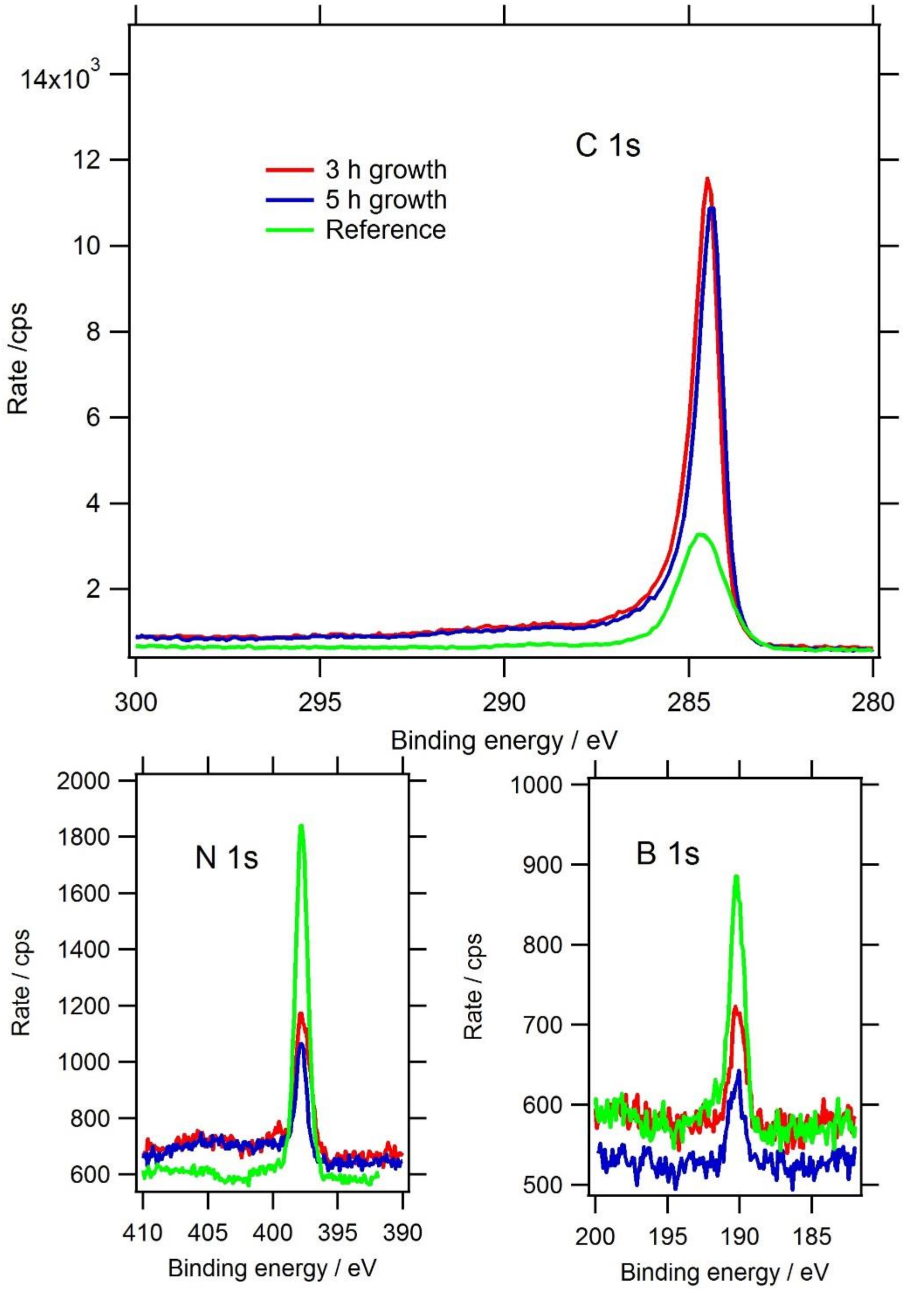

Figure SI-5. Example XP spectra from one area each of the 3h and 5 h deposited samples with atomic carbon source and the reference sample, which was heated but no deposition, C 1s, N 1s and B 1s as labelled. From the C 1s spectra it is clear that the carbon peaks for the two deposited samples are very similar in shape with large asymmetry suggesting sp2 type bonding

dominates, whilst the reference sample with no deposition has a carbon peak with a broader more symmetrical shape suggesting more sp3 bonding. The N 1s and B 1s peaks are less intense on the deposited samples, which can be interpreted to be due to the thicker layer of carbon on top attenuating the photoelectron signal. No obvious differences in the peak shape of B 1s or N 1s are evident, so one cannot infer any changes in the surface chemical state (such as direct reaction with the deposited graphene layer) from these spectra.
